# The hidden burden of influenza: A review of the extra‐pulmonary complications of influenza infection

**DOI:** 10.1111/irv.12470

**Published:** 2017-09-13

**Authors:** Subhashini A. Sellers, Robert S. Hagan, Frederick G. Hayden, William A. Fischer

**Affiliations:** ^1^ Division of Pulmonary and Critical Care Medicine The University of North Carolina at Chapel Hill Chapel Hill NC USA; ^2^ Division of Infectious Diseases The University of Virginia Charlottesville VA USA

**Keywords:** extra‐pulmonary complications, influenza, respiratory virus, viral encephalitis, viral myocarditis

## Abstract

Severe influenza infection represents a leading cause of global morbidity and mortality. Although influenza is primarily considered a viral infection that results in pathology limited to the respiratory system, clinical reports suggest that influenza infection is frequently associated with a number of clinical syndromes that involve organ systems outside the respiratory tract. A comprehensive MEDLINE literature review of articles pertaining to extra‐pulmonary complications of influenza infection, using organ‐specific search terms, yielded 218 articles including case reports, epidemiologic investigations, and autopsy studies that were reviewed to determine the clinical involvement of other organs. The most frequently described clinical entities were viral myocarditis and viral encephalitis. Recognition of these extra‐pulmonary complications is critical to determining the true burden of influenza infection and initiating organ‐specific supportive care.

## 1 INTRODUCTION

Severe acute respiratory viral infections, including influenza, are leading causes of global morbidity and mortality. Each year, influenza infects approximately 10%‐20% of the world's population resulting in 3‐5 million hospitalizations and an estimated 87.1 billion dollars in total annual economic burden in the United States alone.^1^, ^2^, ^3^ The true burden of influenza infection is likely much larger as current estimates are based primarily on the recognition of respiratory symptoms (eg, influenza‐like illness). Epidemiologic investigations and case reports indicate that influenza infection often results in diverse phenotypic presentations including involvement of organ systems other than the respiratory tract. A number of rare, but sometimes severe, syndromes are increasingly being recognized. These extra‐pulmonary manifestations of influenza are likely the result of either unique viral pathogenesis (eg, avian A[H5N1]) or host factors (age, comorbidities, genetic predisposition), or both. Most extra‐pulmonary complications are associated with the acute phase of the infection and often manifest as the presenting symptoms. Others, particularly the post‐infectious central nervous system (CNS) syndromes (eg, Guillain‐Barre syndrome [GBS]) and exacerbations of underlying conditions (eg, ischemic heart disease, cerebrovascular disease) may follow infection by weeks to months. In addition, there remains controversy regarding the possibility of late onset sequelae (eg, Parkinson's disease). Multiple influenza‐associated extra‐pulmonary complications can also occur simultaneously in a single patient.^4^ Improved recognition of these complications will improve the understanding of the morbidity and mortality caused by influenza viruses worldwide and allow for better organ‐specific supportive care to reduce influenza‐associated morbidity and mortality. In an attempt to better understand the burden of extra‐pulmonary complications of influenza infection, a comprehensive literature review was performed.

### 1.1 Search strategy and selection criteria

The authors undertook a comprehensive literature review by querying MEDLINE (PubMed database) for articles in English pertaining to extra‐pulmonary complications of influenza infection published between January 1, 1959, and July 1, 2016, using organ‐specific search terms determined by author consensus (Table 1).

**Table 1:** Description of organ‐specific MeSH search terms used in literature search

Organ system	MeSH search terms used
Cardiac	Arrhythmia, cardiac ischemia, cardiac tamponade, cardiomegaly, cardiomyopathy, coronary artery disease, endocarditis, myocarditis, myocardial infarction, heart arrest, heart failure, heart valve disease, pericardial effusion, pericarditis, pulmonary heart disease
Neurologic	Cerebrovascular accident, encephalopathy, encephalitis, encephalomyelitis, Guillain Barre syndrome, meningitis, Reye syndrome, seizure, stroke, transverse myelitis
Ocular	Conjunctivitis, optic neuritis, retinopathy, uveal effusion
Renal	Acute kidney injury, acute tubular necrosis, glomerulonephritis, Goodpasture, hemolytic uremic syndrome, myoglobinuria, rhabdomyolysis
Musculoskeletal	Myopathy, myolysis, myositis
Hepatic	Hepatitis, hepatic vein thrombus, liver disease, portal vein thrombus, transaminitis
Hematologic	Leukopenia, lymphopenia, thrombocytopenia, disseminated intravascular coagulation, embolism, thrombosis, clot, hemolytic uremic syndrome, thrombotic thrombocytopenic purpura, hemophagocytic syndrome
Endocrine	Diabetes mellitus, diabetic ketoacidosis, hyperglycemic hyperosmolar nonketotic coma

**Figure 1:**
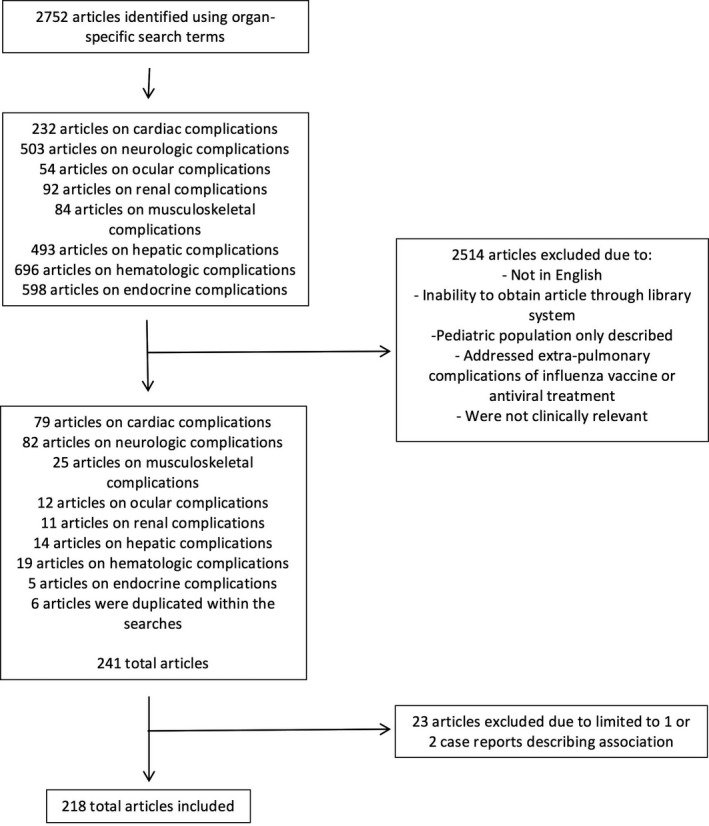
Results of search strategy

Two authors independently reviewed the abstracts for inclusion. Studies that were not in English, included only pediatric populations, focused on extra‐pulmonary complications of influenza vaccines or antiviral therapy were not included (Figure 1). A separate comprehensive review of extra‐pulmonary manifestations in pediatric populations is concurrently being prepared.

The search terms yielded 2752 articles and 2514 were excluded (Figure 1). Two authors independently reviewed 241 articles. For case reports and case summaries, specific data regarding demographics, clinical features, and outcomes were extracted. References of the included articles were also reviewed to ensure complete capture of relevant articles.

## 2 FINDINGS

### 2.1 Cardiovascular complications of influenza

Cardiovascular disease and influenza have long been associated due to an overlap in the peak incidence of each disease during winter months. Epidemiologic studies have also noted an increase in cardiovascular deaths during influenza epidemics indicating that cardiovascular complications of influenza infection, including exacerbation of heart failure, acute ischemic heart disease, and less often acute myocarditis, are important contributors to morbidity and mortality during influenza infection.^5^, ^6^, ^7^ Additionally, studies from vaccine and antiviral therapeutic trials also highlight an important association and suggest that specific pharmacologic strategies may prevent or reduce the risk of many of the cardiovascular complications of influenza.^8^, ^9^, ^10^

#### 2.1.1 Myocarditis

Clinically diagnosed myocarditis, based on a combination of symptoms, elevated cardiac enzymes, and echocardiographic findings, has been reported in approximately 0.4%‐13% of hospitalized adult patients with documented influenza.^11^, ^12^ Myocarditis may, however, be a more common feature in fatal influenza infections as classic histopathologic findings, including cellular infiltration and myocyte necrosis, have been found in 30%‐50% of patients at autopsy despite cardiac involvement not being clinically suspected.^13^, ^14^, ^15^ Additionally, influenza‐related myocarditis often occurs in the absence of more severe respiratory complications as only 40% (6/15) of patients with myocarditis in one study also had documented pneumonia.^16^

There have been approximately 44 cases of influenza‐associated myocarditis in adult patients described in case reports and case series (Table 2).^15^, ^17^, ^18^, ^19^, ^20^, ^21^, ^22^, ^23^, ^24^, ^25^, ^26^, ^27^, ^28^, ^29^, ^30^, ^31^, ^32^, ^33^, ^34^, ^35^, ^36^, ^37^, ^38^, ^39^, ^40^, ^41^, ^42^ Among those reported, 52% (23/44) occurred in men and 68% (30/44) occurred in patients under 40 years of age. Although much of the information on influenza‐associated myocarditis comes from Japan, case reports have been published from all regions including Europe, the Middle East, North America, the Caribbean, and elsewhere in Asia. The association of different influenza strains and subtypes with myocarditis is limited as 31/44 (70%) involved influenza A(H1N1)pdm09. The remaining 12 cases involved influenza B infection (11%; 5/44), A(H3N2) infection (4%; 2/44), and no subtype was reported in 6 others (14%; 6/44).

**Table 2:** Summary of myocarditis cases in the setting of influenza infection reported in the literature (case reports and case series)

Age	Sex	Virus	Cardiac symptoms present at admission	Onset of symptoms^a^	Echocardiographic findings	Elevated cardiac enzymes	Advanced cardiac support	Survival^b^
17	F	A(H1N1)	Yes	Not reported	EF 20%, large effusion	Not reported	ECMO, BiVAD	Y
17	F	A	Yes	3 d	Day 0 with tamponade, normal EF Day 3 EF 30%	Yes	None	Y
21	M	A(H1N1)	Yes	7 d	EF 50%, small effusion	Yes	No	Y
24	F	A(H1N1)	Yes	Not reported	EF 10%‐15%, moderate effusion	No	None	Y
24	M	B	No	Few weeks	Effusion with tamponade	Not reported	None	Y
24	F	A(H1N1)	Yes	6 d	EF 34%, diffuse hypokinesis, effusion	Yes	PCPS, IABP	Y
25	M	A(H3N2)	Yes	10 d	Day 0—normal Day 3—pericardial effusion	Yes	None	Y
25	F	A(H1N1)	Yes	2 d	Reported as myopericarditis	Not reported	None	Y
30	F	A(H1N1)	No	6 d	EF 15%, LV enlargement, moderate effusion with right‐side collapse	Yes	None	Y
30	F	A	Yes	5 d	EF 20%, global hypokinesis	Yes	BiVAD	Y
30	M	A(H1N1)	Yes	10 d	EF 10%	Yes	IABP	Y
31	M	A(H1N1)	No	9 d	Diffuse hypokinesis, effusion	Yes	IABP	Y
31	F	A(H1N1)	Yes	2 d	Severe hypokinesis	Yes	PCPS, IABP	Y
34	F	A(H1N1)	No	1 d	EF 23%, diffuse hypokinesis	Yes	PCPS, IABP	N
34	F	A(H1N1)	Yes	7 d	EF 45% with regional hypokinesia	Yes	No	Y
34	F	B	Yes	10 d	EF 8%, dilated ventricles, hypokinesis, LV thrombus	Yes	IABP, LVAD	N
35	F	A(H1N1)	Yes	0 d	EF 15%	Yes	ECMO	Y
36	M	A(H1N1)	Yes	7 d	EF 20%, global hypokinesis, moderate effusion	Yes	PCPS	N
36	M	A	Not reported	Not reported	Normal	No	No	Y
41	F	B	Yes	4 d	EF 30%, moderate effusion	Yes	IABP, PCPS	N
40	F	A(H1N1)	Yes	10 d	Not reported	Not reported	Transplant, artificial heart	N
43	M	B	Yes	4 d	No	Yes	No	Y
44	M	A(H1N1)	Yes	5 d	Not performed; EF 27% by catheterization	Yes	ECMO	Y
44	M	A(H1N1)	No	21 d	EF 16%	Not reported	None	Y
44	F	A(H1N1)	No	3 d	EF 24%	Yes	PCPS, IABP	Y
47	F	A(H1N1)	Yes	4 wk	EF 15%	Not reported	ECMO, septostomy	Y
48	F	A(H1N1)	Yes	2 d	EF 20%	Yes	None	Y
50	M	A(H1N1)	Yes	2 d	EF 30%, regional hypokinesia	No	No	Y
50	M	A(H1N1)	Yes	3 d	EF 40%, regional hypokinesia, four‐chamber dilation	Yes	None	Y
51	F	A	No	6 d	Pericardial effusion with tamponade	Not reported	None	Y
52	F	B	Yes	6 d	EF 10%	Yes	EBMO	Y
52	M	A(H1N1)	Yes	Not reported	EF 24%	Yes	IABP, PCPS	N
52	M	A(H3N2)	Yes	7 d	EF 28%	Yes	IABP	Y
53	M	A(H1N1)	No	3 d	EF 40%, diffuse hypokinesis	Yes	None	Y
58	M	A(H1N1)	Yes	10 d	EF 30%‐34%, diffuse hypokinesis	Yes	None	Y
60	F	A(H1N1)	Yes	3 d	EF 10% with global hypokinesis and dilated left atrium	Not reported	IABP, PCPS, ECMO	Y
61	M	A(H1N1)	Yes	2 d	EF 20%	Yes	IABP	Y
61	F	A(H1N1)	Yes	2 d	EF 20%, diffuse hypokinesis	Yes	None	Y
64	M	A	Yes	Not reported	EF 24%, left ventricular hypertrophy	Yes	IABP, PCPS	N
66	M	A(H1N1)	No	7 d	Normal	Yes	None	Y
67	M	A(H1N1)	Yes	3 d	EF 20%, global hypokinesis	Yes	IABP	N
69	M	A(H1N1)	No	8 d	EF 29%, diffuse hypokinesis	Yes	PCPS	N
72	M	A(H1N1)	Yes	2 d	EF 38%, diffuse hypokinesis	Yes	None	Y
75	M	A	Yes	Not reported	Global hypokinesis	Yes	None	N

EF, ejection fraction; ECMO, extracorporeal membrane oxygenation; BiVAD, biventricular assist device; PCPS, percutaneous cardiopulmonary support; IABP, intra‐aortic balloon pump; LVAD, left ventricular assist device.

a Onset of cardiac symptoms from initial viral symptoms.

b Survival until hospital discharge.

The clinical course of influenza‐associated myocarditis is variable, with the majority of patients experiencing an acute onset of symptoms related to cardiac dysfunction, including chest pain, dyspnea, syncope, hypotension, and arrhythmia, between days 4 and 7 following the initial symptoms of viral infection.^43^ Most patients, with admission symptoms reported, presented to the hospital with cardiac symptoms (97%; 34/36), suggesting that it was the cardiovascular manifestations that were responsible for the patient seeking medical attention rather than typical respiratory symptoms. Of the 37 cases that documented time to onset of cardiac symptoms, only 3 (8%) developed symptoms late in their course as defined by greater than 10 days after initial viral symptoms.^15^, ^20^, ^36^

The severity of influenza‐associated myocarditis spans a wide spectrum ranging from asymptomatic to severe disease. Although recognition of influenza‐associated cardiovascular complications occurs primarily in patients with cardiac symptoms, there is some suggestion that a significant proportion of patients with influenza infection may suffer clinically unrecognized, asymptomatic myocardial injury. A Japanese study of 96 patients found that 11% of patients who were infected with influenza A (H3N2) had elevated myosin light‐chain I concentrations, a marker for myocardial injury.^44^ However, the degree of injury is likely mild as another study of 152 slightly younger patients in the UK found no elevation of cardiac troponin I and T levels, which are more sensitive markers of cardiac injury.^45^ A third study followed 30 previously healthy young adults diagnosed with influenza infection for 28 days after enrollment with serial electrocardiograms, echocardiograms, and cardiac enzymes and found that no enrolled patients had clinically significant changes in their cardiac studies or cardiac enzymes.^46^

At the other end of the spectrum, a number of cardiac‐specific complications have been described in the setting of influenza‐associated myocarditis including heart failure, arrhythmias, pericardial effusion, and cardiac tamponade. Congestive heart failure, as diagnosed by regional or global hypokinesis on echo/MRI, is the most common complication and is seen in 84% (37/44) of patients with influenza‐associated myocarditis. More than half (23/37; 62%) of patients with heart failure from influenza‐associated myocarditis required advanced cardiac support therapies. Thirty‐eight percent (14/37) were treated with intra‐aortic balloon pumps (IABP), 27% (10/37) with percutaneous cardiopulmonary support, and 16% (6/37) who required extracorporeal membrane oxygenation. Two patients had a biventricular assist device (BiVAD) implanted, one had a left ventricular assist device placed, one had a surgical atrial septostomy performed, and one patient underwent a heart transplant which failed requiring placement of an artificial heart (Jarvik).

Although heart failure associated with influenza can be severe and cause significant hemodynamic compromise requiring advanced cardiac support therapies, recovery of cardiac function has been documented frequently in survivors. Of the 37 patients who were diagnosed with heart failure in the setting of influenza‐associated myocarditis, with ejection fractions ranging from 8% to 50%, 26 (70%) ultimately experienced resolution of their systolic dysfunction on echocardiogram. All of the patients (n = 13) who had time to resolution reported did so within 20 days of the onset of dysfunction.

Pericardial effusions of variable size and significance also frequently complicate influenza‐ associated myocarditis. Thirteen of 44 (30%) patients with influenza‐associated myocarditis had pericardial effusions on echocardiogram, and 4 had evidence of tamponade requiring pericardiocentesis. Other reported complications include eight cases of life‐threatening ventricular arrhythmias and one case of myocardial infarction (MI). Additionally, recurrent myocarditis in the setting of influenza infection has been reported in 2 cases. One patient had two prior episodes of myocarditis and then a third elicited in the setting of influenza infection, consistent with influenza infection exacerbating an underlying condition.^29^ Another patient had resolution of his influenza‐associated myocarditis followed by recurrent myocarditis 22 days after initial presentation.^29^, ^33^

The underlying pathophysiology of influenza‐associated myocarditis remains unclear. In a study of endomyocardial biopsies from 38 patients with viral and non‐viral mediated myocarditis, the viral etiology was determined by PCR in 20 patients including 2 with influenza.^47^ In another study of 29 patients with fatal influenza B infection with cardiac tissue samples available at autopsy, only one patient had influenza B detected by RT‐PCR.^13^ Of the case reports described above, the pericardial fluid of one patient was found to be positive for influenza A(H1N1)pdm09by PCR,.^24^ Another patient had evidence of influenza A(H1N1) in her myocardium identified by both immunofluorescence and viral culture after her heart was replaced by a Jarvik artificial heart on hospital day 4; in this case, influenza A was also detected in her blood.^32^ Evidence of virus in these cases suggests a potential role for direct viral invasion as the underlying pathogenic mechanism, although PCR positivity alone does not provide definitive evidence. Alternatively, the increased incidence of myocarditis among patients with more severe influenza infection may implicate an exaggerated immune response in the pathogenesis, as increased serum cytokines are commonly found in severe influenza.^48^, ^49^, ^50^, ^51^, ^52^ Increasing levels of IL‐8, IL‐10, IL‐6, and TNF‐α were reported in one patient with influenza‐associated myocarditis concurrent with her clinical deterioration supporting the role of systemic inflammation as a driver of influenza‐associated myopericarditis.^53^ Similarly, elevated levels of TNF‐α mRNA and protein were found in endomyocardial biopsies from 16 of 20 (80%) patients with viral myocarditis compared with 3 of 19 (17%) patients with non‐viral etiologies of myocarditis.^47^ Early recognition is critical as many patients required advanced cardiac supportive care, and the mortality among the reported 43 patients with influenza‐associated myocarditis is approximately 23%.

Heart failure independent of myocarditis has also been described in the course of influenza infection. In one series of 124 patients hospitalized with influenza infection, 24 had echocardiograms (echo) performed and 6 (25%) of those patients had new or worsened left ventricular dysfunction.^54^ Four of these patients experienced at least partial improvement in ejection fraction by echo at 4‐22 days. A second retrospective, multicenter study looked at 23 ICU patients with A(H1N1)pdm09 infection and found that right ventricular dysfunction (48%) was more common than left ventricular dysfunction (17%) and was higher in prevalence than generally reported in patients with acute respiratory distress syndrome (ARDS) (10‐25%).^55^

#### 2.1.2 Ischemic heart disease

Several large epidemiologic studies in Russia, the United States, the United Kingdom, and Hong Kong have revealed a temporal association between the circulation of influenza viruses and an increase in hospitalizations and deaths due to ischemic heart disease (IHD).^5^, ^56^, ^57^, ^58^ Additionally, a significant increase in the number of IHD deaths has also been detected during epidemic periods.^57^ Two large studies utilizing self‐controlled case series analysis found that rates of a first MI were highest in the first 3 days following an acute respiratory infection with a reduction in the effect over time.^6^, ^58^ Another study of 600 patients in the Veterans Administration system found that 143 (24%) patients who tested positive for influenza had acute cardiac injury, 80% of which occurred within 3 days of the influenza diagnosis and 70 (49%) were MIs. The remaining half had elevations in cardiac biomarkers due to acute congestive heart failure (8/143; 6%), myocarditis (6/143; 4%), atrial fibrillation (4/143; 3%), non‐cardiac explanations (11/143; 8%), or no documented explanation (44/143; 31%).^59^ In contrast, only 13 (3.6%) of 2287 patients diagnosed with an unspecified community‐acquired pneumonia (CAP) were diagnosed with MI in the 30 days after CAP diagnosis.^60^

Given the association between peak influenza activity and cardiovascular events, studies have evaluated the effect of influenza vaccination on the reduction of multiple cardiovascular end points (Table 3). The incidence of acute MI was significantly reduced in the 60 days following influenza vaccination in one self‐controlled case series of the inactivated influenza vaccine.^61^ This effect was most pronounced in the first 2 weeks after vaccination with a reduction of acute MI by 32% (incidence rate ratio, IRR, 0.68; 95% CI 0.60‐0.78) and decreased to 18% (IRR 0.82; 95% CI 0.75‐0.90) at 25‐59 days post‐vaccination. Similarly, a larger prospective randomized double‐blinded placebo controlled study of 658 patients with coronary artery disease (CAD) found a significant reduction in coronary ischemic events, specifically cardiovascular death, MI, cardiac revascularization, or hospitalization with myocardial ischemia, in the 12 months following influenza vaccination with the inactivated influenza vaccine compared to subjects receiving a placebo vaccine (HR 0.54; 95% CI 0.29‐0.99).^62^ A case‐control trial in the UK evaluated over 75 000 patients for an association between influenza vaccination and the diagnosis of a first acute MI between 2001 and 2007 and found that vaccination within the previous year was associated with a lower rate of acute MI (OR 0.81; 95% CI 0.77‐0.85).^63^

**Table 3:** Summary of studies evaluating effect of anti‐influenza vaccination or treatment in ischemic heart disease

Study	Study type	Intervention	End points	Event rate in unvaccinated subjects	Event rate in vaccinated subjects	
Gwini et al	Self‐controlled case series	Influenza vaccination within 14 d 25‐59 d post‐vaccination	Incidence of acute MI			Incidence rate ratio 0.68 (95% CI 0.60‐0.78) Incidence rate ratio 0.82 (95% CI 0.75‐0.90)
Ciszewski et al	Randomized double‐blind placebo‐controlled	Influenza vaccination 12 mo prior	Coronary ischemic event	9.97%	6.02%	Hazard ratio 0.54 (95% CI 0.29‐0.99)
Siriwardena et al	Matched case control	Influenza vaccination within the previous year	Incidence of first acute MI	52.9%	51.2%	Odds ratio 0.81 (95% CI 0.77‐0.85)
Gurfinkel et al	Randomized, single‐blind	Influenza vaccination 6 mo prior	Composite CV death, non‐fatal MI, severe recurrent ischemia	23%	11%	Relative risk 0.50 (95% CI 0.29‐0.85)
Naghavi et al	Case control	Influenza vaccination within the same influenza season	New MI after initial MI	71%	47%	Odds ratio 0.33 (95% 0.13‐0.82)
Jackson et al	Cohort	Influenza vaccination within the same season	New MI after initial MI			Hazard ratio 1.23 (95% CI 0.81‐1.87)

Study	Study type	Intervention	End points	Event rate in untreated subjects	Event rate in treated subjects	
Casscells et al	Cohort	Oseltamivir prescribed	Recurrent CV diagnosis within 30 d of influenza diagnosis	21.2%	8.5%	Odds ratio 0.417 (95% CI 0.35‐0.50)

MI, myocardial infarction; CV, cardiovascular.

A number of studies evaluating the impact of influenza vaccination on subsequent cardiovascular events have yielded mixed results. In the FLUVACS study, the 100 subjects with a new MI who were randomized to receive an inactivated influenza vaccine experienced a significant decrease in the composite end point of cardiovascular death, non‐fatal MI, or severe ischemia at 6 months (RR 0.50; 95% CI 0.29‐0.85) compared with those who received placebo.^8^ Similarly, a case‐control study of 218 patients with CAD in the United States demonstrated that the influenza vaccine was protective against a new MI in subjects who had already experienced an MI (OR 0.33; 95% CI 0.13‐0.82).^9^ However, a separate retrospective study of 1378 patients who had survived a prior MI found no association between influenza vaccination and reduction in a subsequent MI.^64^ Taken together, these studies strongly suggest that influenza vaccination is associated with a reduction in *initial* MIs, while larger studies on secondary prevention are needed.

Only one retrospective study has evaluated the effect of neuraminidase inhibitors on the recurrence rate of cardiovascular disease following influenza infection.^10^ Patients with a prior history of cardiovascular disease and acute influenza infection were prescribed oseltamivir and those who filled with prescription within 2 days suffered fewer recurrent MIs (0.2% vs 1.4%; *P* < .005) and less combined cardiac events, including MI, angina, stroke, heart failure, and sudden cardiac death compared with untreated patients within 30 days of influenza diagnosis (OR 0.417; 95% CI 0.35‐0.50). The low percentage of people who filled their prescription within 2 days (18.2%) represents a potentially important missed opportunity to reduce cardiovascular complications from influenza infection.

Influenza‐associated IHD is posited to be driven by inflammation which is known to have a critical role in the development of acute coronary syndrome.^65^ In apolipoprotein E‐deficient mice, an animal model of atherosclerosis, animals that were infected with influenza A virus developed subendothelial and smooth muscle inflammatory infiltration with overlying platelets and fibrin strands in atherosclerotic plaques.^66^ Additionally, the systemic pro‐inflammatory response triggered by influenza infection is accompanied by significant pro‐coagulant effects which may also play a role.^67^

Viremia in influenza infection has been reported variably—one study of 139 patients with influenza A(H1N1)pdm09 infection reported viral RNA detected by RT‐PCR in 14 patients (10%), which was associated with severe clinical disease and presence of the D222G/N mutation in the viral hemagglutinin protein.^68^ Earlier studies in non‐pandemic influenza strains identified viremia in up to 17% of patients.^69^ At present, no human studies support the theory of direct virus invasion of the vascular bed. However, influenza A virus has been detected in the atherosclerotic arteries of apolipoprotein E‐deficient mice that were infected, suggesting that this is a possibility.^70^ There is also limited evidence, however, that prior infection may play a role in the development of chronic atherosclerosis. One cross‐sectional study of patients referred for cardiac catheterization found that while influenza IgG seropositivity is not independently associated with IHD alone, patients who were seropositive for multiple pathogens were more likely to have IHD suggesting that cumulative infection burden may be related to development of atherosclerosis.^71^

#### 2.1.3 Stroke

Similar to ischemic cardiac complications, the risk of ischemic cerebral vascular accidents (CVA), or strokes, appears to be significantly increased in the days after a respiratory tract infection.^58^ However, only two cases of ischemic CVA in the setting of influenza infection have been reported. One case of multiple strokes occurred in a young woman with disseminated intravascular coagulation who was critically ill.^72^ The other case occurred in a 50‐year‐old woman with ARDS secondary to influenza A(H1N1) who developed multiple strokes in the territory of the right middle cerebral artery.^73^ There may be indirect evidence of an association between influenza and CVAs, but data from influenza vaccination and neuraminidase inhibitors are conflicting.^74^, ^75^ One case‐control study in France found that the risk of stroke was reduced in subjects vaccinated in that year compared to those not vaccinated (OR 0.50, 95% CI 0.26‐0.94). Protection was strongest in patients under the age of 75.^76^ Similarly, another study in Germany also found a reduced risk for stroke with influenza vaccination (OR 0.46, 95% CI 0.28‐0.77).^77^ When stratified by type of stroke, the reduced risk was most significant for ischemic stroke with a trend toward protection in hemorrhagic stroke and no protection against transient ischemic attacks (TIA). In contrast, a large study of over 23 000 patients from two prospective cohorts (OPTIC and ARMISTAD) and a randomized trial (PERFORM) did not find any association between influenza vaccination and cerebrovascular accidents, although patients in this study had higher levels of known CVA risk factors including dyslipidemia and hypertension.^78^ In this study, propensity score matching was used to compensate for potential healthy user bias that may explain the significant benefit seen in previous studies. Lastly, there is evidence that treatment with the neuraminidase inhibitor oseltamivir in the setting of acute influenza infection reduces the risk of stroke.^79^ The protective effect remained significant in patients under the age of 65 for 6 months after infection, whereas in patients 65 and older, the association was significant only in the first month after infection.^79^

Cardiac complications associated with influenza infection, including myocarditis, ischemic heart disease, and stroke, can have a significant impact on influenza mortality and morbidity. Early recognition of these complications is critical to initiate organ‐specific supportive care Additionally, the protective role of influenza vaccines against an initial MI and early initiation of neuraminidase inhibitor with a decrease in combined cardiovascular end points highlights important preventative and therapeutic opportunities to reduce influenza‐associated cardiovascular complications.

### 2.2 Neurologic complications of influenza

Influenza infection can lead to a variety of neurologic complications including a number of specific clinical entities grouped together as influenza‐associated encephalitis or encephalopathy (IAE), as well as a separate syndrome known as post‐influenza encephalitis, GBS, Reye's syndrome, and Parkinsonian symptoms. While neurologic complications are more frequently noted in pediatric populations, they are also increasingly being recognized in adults.^80^ The neurologic manifestations of influenza have been primarily reported in Japan, which may be due to greater recognition in that region. However, a study of influenza‐associated neurologic complications in the USA did find that a disproportionate number of Asian/Pacific Islanders were affected (12.79/1 000 000) compared with white, non‐Hispanic patients (3.09/1 000 000) suggesting a possible underlying genetic predisposition.^81^

#### 2.2.1 Influenza‐associated encephalitis/encephalopathy

Influenza‐associated encephalopathy or encephalitis (IAE) is a rapidly progressive encephalopathy primarily characterized by an impaired level of consciousness developing within a few days of influenza infection. A number of distinct clinical syndromes have been described, predominantly in the pediatric literature, and fall under the umbrella category of IAE including acute necrotizing encephalopathy (ANE), acute encephalopathy with biphasic seizures and late reduced diffusion (AESD), and mild encephalitis/encephalopathy with reversible splenial lesion (MERS). ANE is often a fulminant complication characterized by multiple brain lesions frequently involving the thalami. AESD is characterized by a biphasic course, febrile seizures, and subcortical white matter lesions on MRI, whereas MERS, which is characterized typically by a lesion of the splenium of the corpus callosum, is associated with a milder course and often a good clinical outcome.^82^ The spectrum of influenza‐associated encephalitis can also be expanded to include posterior reversible encephalopathy syndrome (PRES), which is associated with areas of edema on MRI and can occur days to weeks after initial viral symptoms, and acute hemorrhagic leukoencephalopathy (AHLE), characterized by a rapid and fulminant demyelination and inflammation of the white matter. However, these entities are primarily described in the pediatric literature as IAE is more frequently recognized and reported in children less than 5 years of age. The incidence of IAE in adults has been reported in up to 4% of hospitalized adults, although case reports rarely apply the terminology of specific clinical syndromes beyond IAE.^83^

There have been 28 reported cases of IAE in adults with 7 further differentiating the clinical syndromes including 3 cases of PRES, 2 cases of MERS, and 2 cases of AHLE (Table 4).^4^, ^84^, ^85^, ^86^, ^87^, ^88^, ^89^, ^90^, ^91^, ^92^, ^93^, ^94^, ^95^, ^96^, ^97^, ^98^, ^99^, ^100^, ^101^, ^102^, ^103^, ^104^ In these reports, men are more often affected (19/28; 68%) and patients range in age between 20 and 86 years old. The neurologic symptoms appear early in the disease course, within the first week of viral illness in 22 of 25 (88%) cases that reported the time of onset. Most patients (20/25; 80%) presented to the hospital with neurologic symptoms. Almost all patients (25/27, 93%) experienced symptoms of decreased consciousness, and 37% (10/27) had witnessed seizure activity. Other less common presenting symptoms included urinary retention, vision loss, hemiplegia, cerebellar signs, and opisthotonus. Of the 28 cases of adult IAE reported, 17 (61%) were in the setting of influenza A(H1N1) infection, 4 (14%) were attributable to influenza A(H3N2) infection, 3 (11%) occurred during influenza B infection, and 4 (14%) were secondary to unspecified influenza A viruses.

**Table 4:** Summary of influenza‐associated encephalitis cases reported in the literature (case reports and case series)

Age	Sex	Virus	Subtype	Neurologic symptoms present at admission	Onset of symptoms^a^	Seizure Present	Abnormal imaging	Lumbar puncture findings	Treatment	Survival^b^
20	F	A(H1N1)	NA	No	24 d	No	MRI with posterior parietal and occipital signal abnormalities	Elevated protein	Oseltamivir	Y
20	M	A(H1N1)	NA	Yes	6 d	Yes	CT with diffuse edema, MRI with white matter hyperintensity	Pleocytosis, elevated protein	Oseltamivir, peramivir, dexamethasone	Y
21	F	A	PRES	No	24 d	Yes	CT with unilateral PRES, MRI with bilateral T2 signal abnormalities	Not reported	None	Y
26	M	A(H1N1)	MERS	Yes	Not reported	No	MRI with hyperintense signal on splenium of corpus callosum	Pleocytosis	Oseltamivir, methylprednisolone	Y
26	M	A(H1N1)	NA	Yes	7 d	Yes	CT with sinus thrombosis, 3 cerebral hematomas	Hemorrhage	Not reported	N
27	M	A(H1N1)	AHLE	No	7 d	No	Not reported	Not reported	Oseltamivir	N
27	M	A(H3N2)	NA	Yes	1 d	Yes	CT with low attenuation areas in both thalami, MRI with edema and high signal lesions in thalami, brainstem, and deep white matter	No	Antiviral	Y
31	F	B	NA	Not reported	Not reported	No	No	Influenza +	Not reported	Y
35	M	A	NA	Yes	Not reported	Not reported	Not reported	Not reported	None	N
40	M	A(H1N1)	AHLE	No	30 d	No	CT with b/l subcortical hypodensities with hemorrhage, MRI with multiple b/l lesions with edema	Elevated protein	Oseltamivir, PLEX, methylpred	Y
40	M	A(H1N1)	NA	Yes	2 d	Possible	CT with lesions on vertex, MRI with bilateral frontal hyperintensity	Pleocytosis	Antiviral	Y
45	M	B	NA	Yes	4 d	Yes	No	No	Oseltamivir	Y
46	F	A(H1N1)	NA	No	4 d	Yes	CT with edema	Influenza +	Oseltamivir	Y
46	M	A(H1N1)	NA	Yes	4 d	No	MRI wit bilateral hyperintense lesions in T1 images	No	Oseltamivir	N
46	F	A(H1N1)	NA	Yes	3 d	No	No	No	Oseltamivir	Y
51	M	A(H3N2)	MERS	Yes	Several days	No	MRI with diffusion restricted lesion in the splenium of the corpus callosum	Not reported	None	Y
51	M	A(H1N1)	PRES	No	1 d	Yes	MRI with increase signal in L mesial frontal cortex, hypoperfusion of L fronto‐temporal region	No	None	Y
51	M	A	NA	Yes	2 d	No	Not reported	Pleocytosis	Oseltamivir	Y
51	M	A(H1N1)	NA	Yes	1 d	Yes	No	Influenza +	Oseltamivir	N
53	M	A(H1N1)	NA	No	5 d	No	Not reported	Influenza +	Oseltamivir	N
55	M	A(H1N1)	NA	Yes	1 d	Yes	No	No	Oseltamivir	Y
60	F	A(H1N1)	NA	Yes	4 d	No	MRI with diffuse T2 signal abnormalities	Influenza +	Oseltamivir	Y
61	F	B	NA	Yes	3 d	No	No	Elevated protein	Oseltamivir	Y
65	F	A	PRES	Yes	3 d	No	MRI with signal abnormalities in b/l parietal, occipital, and cerebellar hemispheres	No	Oseltamivir	Y
71	M	A(H1N1)	NA	Yes	Few days	Yes	CT with slight vascular degeneration	No	Oseltamivir	Y
72	M	A(H3N2)	NA	No	3‐4 d	Possible	No	No	Oseltamivir	Y
76	M	A(H1N1)	NA	Yes	1‐2 d	No	No	No	None	Yes
86	F	A(H3N2)	NA	Yes	1‐2 d	No	CT with old ischemic changes and a meningioma	No	Oseltamivir	Yes

MRI, magnetic resonance imaging; CT, computed tomography; PRES, posterior reversible encephalopathy syndrome; MERS, mild encephalopathy/encephalitis with reversible splenial lesion; AHLE, acute hemorrhagic leukoencephalopathy.

a Onset of neurologic symptoms from initial viral symptoms.

b Survival to hospital discharge.

In 18 cases with CT scans reported, 7 (39%) had acute abnormalities including 3 (17%) with patchy hypodense lesions on the vertex and the in the thalamus, 2 (11%) with diffuse edema, 1 (5%) with focal left parietal and occipital cortical edema, and 1 (5%) with superior sagittal thrombosis and cerebral hematomas. Nine (50%) adult patients with IAE had normal CT scans, and the remaining two patients (11%) had evidence of chronic changes. Of the 10 patients with MRI results reported, all were abnormal, primarily involving signal abnormalities throughout the cortex, white matter, or brainstem. Three patients (30%) were diagnosed with PRES based on findings of vasogenic edema in the frontal cortex in one and in the parietal and occipital cortex in the other two; only one of these patients had follow‐up MRI imaging which demonstrated resolution at day 24.^4^, ^91^, ^101^ Two patients’ MRIs had the hyperintense signal on the central splenium of the corpus callosum typical of MERS; follow‐up MRI at day 11 was normal in the only patient with follow‐up imaging reported.^98^, ^104^ Electroencephalography (EEG) testing revealed diffuse slowing typical of encephalitis in 11/14 (79%), epileptic discharges in 1/14 (7%), and was normal in 1/14 (7%).

Lumbar puncture (LP) was performed in 24 of 28 patients and demonstrated a pleocytosis in 4 cases (17%), elevated protein in 4 cases (17%), and was normal in 11 cases (46%). RT‐PCR was positive for influenza in 5 of the 24 (21%) patients that had a lumbar puncture (LP) performed.^92^, ^94^, ^95^ None of these patients received systemic corticosteroids prior to the LP. Similarly, the post‐mortem examination of two patients that died of IAE yielded viral RNA isolated from the brain; however, there was no discussion of whether the location of the viral RNA correlated to imaging findings.^89^, ^90^

All patients were treated with supportive care, and 21 of the 28 patients reported were treated with neuraminidase inhibitors including oseltamivir, peramivir, and/or zanamivir. Three patients (11%) were additionally treated with corticosteroids, and one patient underwent plasma exchange.^85^, ^87^, ^98^ Overall, 79% (22/28) of adult patients with IAE survived. However, 25% (7/28) of patients suffered residual neurologic defects, primarily including motor weakness and cognitive defects at follow‐up within 3 months of the initial illness. Long‐term follow‐up beyond 3 months has not been reported.

A separate entity, post‐influenza encephalopathy has also been described, in which the development of neurologic symptoms occurs after the resolution of the respiratory symptoms but within 3 weeks of the influenza diagnosis.^83^ One case report described post‐influenza encephalopathy, including altered mental status, seizures, involuntary movements, and cortical blindness that developed 3‐4 weeks following a severe respiratory disease with influenza A(H1N1) virus infection.^105^ The patient's MRI demonstrated diffuse multifocal lesions with both gray and white matter. In a study of 20 patients with encephalopathy in the setting of influenza, fourteen patients were classified as having post‐influenza encephalopathy, with development of neurologic symptoms at a median of 9.5 days (range, 7‐23 days).^83^ Of the twelve patients who had MRIs performed, six (50%) had MRI abnormalities which demonstrated demyelination, suggesting a different pathophysiology of late‐developing neurologic symptoms in which immunologic mechanisms are likely operative.

The pathogenesis of influenza‐associated encephalopathy and encephalitis in adults remains undefined. Demonstration of viral RNA, as detected by rRT‐PCR, in brain tissue and CSF suggests direct viral invasion of the CNS.^106^, ^107^, ^108^, ^109^ Patients with IAE more frequently experience concurrent hepatic and renal function dysfunction, which could suggest a component of metabolic encephalopathy coexisting with severe influenza illness rather than as a direct effect of the virus itself.^83^ A dysregulated immune response has also been posited to drive neurologic complications in influenza. Serum levels of cytokines IL‐6, TNF‐alpha, and IL‐10 were found to be significantly elevated in pediatric patients with IAE as compared to influenza‐infected patients without neurologic involvement.^110^, ^111^ Similarly, CSF levels of IL‐6 were also found to be significantly elevated in pediatric patients with IAE compared to children with neurologic disorders and not infected with influenza.^112^ Transcriptomic profiling has also demonstrated elevated levels of IL‐6, IL‐10, and TNF‐alpha mRNA in patients with IAE compared to those with influenza‐associated febrile seizures in the setting of equivalent viral loads.^111^ One study demonstrated significantly greater serum levels of soluble CD163, a scavenger receptor for hemoglobin‐haptoglobin complexes expressed by monocytes and macrophages in patients with more severe forms of IAE such as ANE compared to those with milder disease suggesting a dose‐response association.^113^

Although IAE has been described in case reports from around the world, there appears to be a higher incidence in East Asian populations, suggesting the possibility of a genetic predisposition. In a study of 29 Japanese patients with AESD or ANE, a higher frequency of several single nucleotide polymorphisms in the carnitine palmitoyl transferase II gene was found compared with healthy controls.^114^ Another study identified an association between AESD and a genetic variant of the adenosine A2A receptor (ADORA2A) which may alter cyclic AMP signaling.^115^ Finally, missense mutations in the gene encoding Ran binding protein 2 (RANBP2) have been identified in a Taiwanese family in which 16 family members were diagnosed ANE.^116^ RANBP2 is a ubiquitin ligase that mediates nuclear transport of HIV pre‐integration complexes and represses replication of Japanese encephalitis virus in vitro [REFS]; these studies imply that altered viral processing may allow a highly adapted respiratory virus to become active in the CNS.^116^, ^117^, ^118^, ^119^ However, much of the data supporting a role for host genetics or a dsyregulated immune response in IAE comes from studies in pediatric populations. Further studies in adults are needed to determine the role of host response in the pathogenesis of IAE.

#### 2.2.2 Guillain‐Barre syndrome

Guillain‐Barre syndrome is an acute immune‐mediated polyneuropathy characterized by progressive ascending symmetric muscle weakness and accompanied by absent deep tendon reflexes. Most cases of GBS are thought to represent an autoimmune response triggered by an infectious agent with onset of symptoms within 2‐6 weeks of the initial infection, most commonly with Campylobacter jejuni, Mycoplasma pneumoniae, or Epstein‐Barr virus. However, 60%‐70% of cases of GBS in Western countries do not have a clear etiology identified, although influenza has been proposed as an additional causative agent.^120^

Guillain‐Barre syndrome secondary to influenza infection was first described in 1959, and five adult cases have been reported since.^121^, ^122^, ^123^, ^124^ Two of the 5 patients (40%) were women, and 4 of 5 (80%) were under the age of 40. All cases of influenza‐associated GBS occurred in the setting of influenza A virus infection, and 40% (2/5) were from pandemic H1N1. In contrast to other infectious agents, the majority of patients with influenza‐associated GBS developed neurologic symptoms within 1 week of the onset of influenza‐like illness. The fifth patient experienced a delayed onset of GBS symptoms approximately 5 weeks after influenza infection.^121^ Of the 5 cases of influenza‐associated GBS, two patients received treatment with IVIG and survived with resolution of their symptoms by 1 month; one patient was treated with plasma exchange and survived although it was not reported whether symptoms resolved or not. Two patients did not receive any disease‐modifying treatment and both died.^124^

Epidemiologic studies that have evaluated an association between preceding influenza and GBS suggest that influenza may be an important and under‐recognized etiology of GBS.^125^, ^126^, ^127^ In a nested case‐control study in the UK, researchers found that influenza‐like illness carried an 18‐fold increase risk for GBS in the 2 months following infectious symptoms (OR 18.6 95% CI 7.5‐46.4).^125^ Similarly, there is a temporal association between influenza infection and GBS with an increased relative incidence of GBS within 30 days (16.64 95% CI 9.37‐29.54) and 90 days (7.35 95% CI 4.36‐12.38) following presentation with an influenza‐like illness.^128^ In a study of 405 French patients with GBS, peaks in the incidence of cases without an identified etiologic agent are also temporally associated 1‐2 months following peaks in the incidence of influenza‐like illnesses.^120^ In an additional analysis of 73 patients with GBS without a clear etiology in the same study, 10 (13.7%) had serologic evidence of recent influenza A infection and 4 (5.5%) with recent influenza B infection.

Although the 1976 influenza swine flu vaccination program was stopped prematurely due to the increased development of GBS in subjects who received this particular vaccine, recent studies of seasonal influenza demonstrate a safe reduction in GBS in vaccinated individuals. Evidence from a study in the UK demonstrated no increased risk of GBS associated with the seasonal influenza vaccine.^129^ Similarly, a Canadian study using universal healthcare system databases to examine the risk of GBS after exposure to the seasonal trivalent inactivated influenza vaccine found a lower attributable risk of admission for GBS (1.03 per million healthcare encounters for influenza) for those vaccinated compared with unvaccinated individuals (17.2 per million healthcare encounters for influenza).^130^ Taken together, these studies demonstrate that while there may be an increase in GBS associated with influenza infection, vaccination appears to be protective.

While the mechanism underlying influenza‐associated GBS is not known, evidence from *C. jejuni*‐induced GBS suggests that molecular mimicry may play a role as the *C. jejuni* lipopolysaccharide induces an antiganglioside antibody response resulting in the immune‐mediated polyneuropathy of GBS. However, of the five cases of influenza‐associated GBS reported, only one patient had detectable antiganglioside antibodies.^121^ Additionally, in a study of 23 patients infected with influenza A(H1N1)pdm09, only one patient tested weakly positive for one antiganglioside antibody, anti‐GM1, and none of the influenza‐infected patients were diagnosed with GBS.^131^

Closely related to GBS are the neuro‐inflammatory demyelinating diseases, acute disseminated encephalomyelitis (ADEM) which affects both the brain and spine and transverse myelitis which affects the spine. Both conditions can occur as post‐infectious complications and influenza have been implicated as an etiologic agent. There have been six cases of ADEM, and one case of transverse myelitis in adults after influenza infection reported in the literature.^105^, ^132^, ^133^, ^134^, ^135^, ^136^ In these cases, all patients presented with focal myasthenia and paresthesia. Some patients also had areflexia or hypotonia and 3/7 (43%) had urinary retention. In all cases, MRI imaging demonstrated characteristic T2 hyperintense lesions throughout the brain and/or spinal cord. Post‐infectious ADEM and transverse myelitis are thought to represent an autoimmune process triggered by the infection that is hypothesized to share antigentic features with antibodies directed at myelin proteins. One patient with influenza‐associated transverse myelitis was found to have high titers of myelin oligodendrocyte glycoprotein antibodies, suggesting a pathogenic role.^137^ Additionally, one patient had recurrent ADEM after a second influenza infection 6 months after his first episode and another had recurrent ADEM after an influenza vaccination suggesting a role for genetic predisposition or formation of an immunologic mechanism.^132^, ^135^

#### 2.2.3 Other neurologic complications of influenza infection

Narcolepsy is a sleep disorder characterized by excessive daytime sleepiness with nighttime disturbance and can be associated with cataplexy, the sudden loss of muscle tone triggered by strong emotions. It is caused by loss of neurons in the hypothalamus that secrete hypocretin, neuropeptides involved in regulation of arousal and wakefulness. However, upper respiratory infections have also been loosely linked to disease presentation given a temporal association between infection and narcolepsy symptom onset, including Streptococcus pyogenes infection.^138^ A retrospective analysis of the onset of narcolepsy in 629 patients in China demonstrated a threefold increase in the incidence of narcolepsy after the 2009 H1N1 influenza pandemic.^139^ In this study, 96% of patients who developed narcolepsy following the pandemic did not report a prior H1N1 vaccination, suggesting that the association was with the virus itself as opposed to the vaccine.

Narcolepsy has long been thought of as a T‐cell‐mediated autoimmune disease given its association with the HLA‐DQB1*06:02 genotype.^138^ However, a recent study of a mouse model lacking B and T cells demonstrated sleep patterns consistent with narcolepsy following infection with influenza A(H1N1) virus.^140^ By week 4 following infection, viral antigens could be detected in the lateral hypothalamus along with loss of up to half of the hypocretin‐secreting neurons in some brains, suggesting direct viral invasion as a potential mechanism. However, the relationship between influenza infection and the development of narcolepsy in humans remains unclear.

Reye's syndrome, a rapidly progressive disease characterized by encephalopathy and fatty infiltration of the liver, has been associated with viral infections including influenza treated with acetylsalicylic acid. Although this was predominantly seen in children, adult cases were reported in the 1970s and 1980s.^141^, ^142^ Since the recognition of the role of acetylsalicylic acid in Reyes syndrome, there has been a steep decline in the incidence of reported cases due to the avoidance of aspirin in the treatment of viral infections.^143^

Encephalitis lethargica (EL) is a neuropsychiatric disorder characterized by sleep disturbances, lethargy and symptoms of basal ganglia dysfunction, or post‐encephalitic Parkinsonism, which includes movement disturbances, abnormal gait, increased muscle tone, and tremor, first described during the 1918 Spanish flu epidemic. Given the temporal association with the 1918 epidemic, influenza was thought to be the causative agent for EL. Later neuropathologic studies of brain tissue from patients with EL during the 1918 epidemic found no evidence of viral antigen.^144^, ^145^ There have been no further EL epidemics since the 1920s although sporadic cases are reported, often following pharyngitis but without documented influenza infection.^146^, ^147^ One early study did identify the presence of influenza A viral particles by immunofluorescence in the brains of six patients who died with non‐epidemic post‐encephalitic Parkinsonism, but no evidence of influenza was found in the brains of 5 patients with idiopathic Parkinson.^148^ Additionally, another study did demonstrate the presence of influenza A viral particles by immunofluorescence in the substantia nigra pars compacta of post‐mortem brains from 5 cases of Parkinson disease and 7 cases of Lewy body dementia but this finding has yet to be replicated.^149^ The relationship between encephalitis lethargica, Parkinson disease, and influenza infection remains unclear. However, a population based case‐control study of patients with Parkinson disease (PD) and Parkinson symptoms without a firm diagnosis (PS) found that recent influenza infection in the last 29 days was associated with development of PS (OR 3.03, 95% CI 1.94‐4.74) but not with PD supporting the idea that influenza infection is associated with transient neurologic sequelae as seen in many of the complications discussed above.^150^

The neurologic complications of influenza infection are extremely variable in presentation and strength of association. In particular, IAE is poorly described in adults and requires MRI for diagnosis in 50% of cases despite being associated with death in 21% of reported cases. Low threshold for diagnostic evaluation and early initiation of antiviral treatment and supportive care remains critical.

### 2.3 Musculoskeletal complications of influenza

While myalgias are a common complaint among individuals with many viral infections, the development of rhabdomyolysis represents a less common but more serious complication. In cases of virus‐associated rhabdomyolysis, influenza is identified as the most common etiology.^151^, ^152^

There have been approximately 27 reported cases of rhabdomyolysis in the setting of influenza infection, although this likely represents a small fraction of the total cases as myositis and/or rhabdomyolysis have infrequently been reported prior to the 2009 A(H1N1) pandemic (Table 5).^4^, ^22^, ^152^, ^153^, ^154^, ^155^, ^156^, ^157^, ^158^, ^159^, ^160^, ^161^, ^162^, ^163^, ^164^, ^165^ Among 18 patients in Mexico with influenza A(H1N1)pdm09 infection, 62% of patients had mild‐to‐moderate elevations of creatinine kinase (CK).^166^ Of the 27 patients reported with influenza‐associated rhabdomyolysis, 15 (55%) were women and 12 (44%) were over the age of 60. The majority of these patients were infected with influenza A as 37% (10/27) were positive for A(H3N2), 14% (4/27) were positive for A(H1N1), and 40% (11/27) had an unspecified influenza A virus. Only two patients (7%) were infected with influenza B.

**Table 5:** Summary of cases of myositis in the setting of influenza infection reported in the literature (case reports and case series)

Age	Gender	Virus	Onset of symptoms^a^	CPK^b^	Renal failure?	RRT	Survival^c^
19	M	A(H1N1)	Not reported	1715 U/L (43‐156)	Yes	No	N
20	M	A	3 d	8413.35 mkat/L	Yes	Yes	Y
21	F	A	3 d	213 000 IU/L	Yes	Yes	Y
25	M	A(H3N2)	10 d	5555 U/L	Yes	No	Y
28	F	A	1 wk	>3000 μ/L (10‐120)	Yes	Yes	Y
28	F	A(H1N1)	Few days	1371 U/L (43‐156)	Yes	No	Y
28	F	A(H1N1)	1 wk	27 820 U/L (13‐156)	No	No	Y
31	F	A	4 d	>100 000 IU/L (<200)	Not reported	Not reported	Y
37	M	A	1 wk	3100 ukat/L	Yes	Yes	Y
44	M	B	4 d	74 550 U/L	Yes	Yes	Y
47	M	B	7 d	157 IU/mL	No	No	Y
47	M	B	Not reported	218 IU	Possible	No	Y
53	F	A	1 wk	20 000 μ/L (10‐120)	Yes	Yes	N
57	F	A	5 d	203 U/L	Yes	Yes	Y
59	M	A(H1N1)	1 wk	>41 000 U/L	Yes	Yes	Y
60	F	A(H3N2)	6 d	4221 IU/L	Not reported	No	Y
65	M	A	1 wk	17 739 IU/L (<170)	Yes	Yes	Y
69	M	A	1 d	810 μkat/L	Yes	Yes	Y
70	F	A(H3N2)	2 d	4432 IU/L (<40‐180)	No	No	Y
74	M	A(H3N2)	3 d	1365 U/L	Yes	Yes	N
75	F	A(H3N2)	2 d	1198 IU/L (<40‐180)	No	No	Y
76	F	A(H3N2)	1 d	1138 IU/L (<40‐180	No	No	Y
76	F	A	1 wk	35 000 U/L (10‐120)	Yes	Yes	Y
77	M	A(H3N2)	7 d	3827 IU/L	Not reported	No	Y
78	F	A(H3N2)	3 d	25 832 IU/L (<40‐180	No	No	Y
82	F	A(H3N2)	2 d	2405 IU/L (<40‐180)	No	No	Y
86	F	A(H3N2)	2 d	9829 IU/L (<40‐180	No	No	Y

RRT, renal replacement therapy.

a Onset of muscular symptoms from initial viral symptoms.

b Values are not standardized units, institutional normal range reported in parenthesis when available.

c Survival to hospital discharge.

The onset of myopathy related symptoms typically begins early in the course of infection, with 93% (25/27) of patients reporting symptoms within 1 week of the onset of respiratory symptoms. Seventy percent (19/27) presented with muscle tenderness, and 33% (9/27) presented with weakness or inability to stand. As myalgias are a common symptom in viral illness, the diagnosis of rhabdomyolysis relies on evidence of muscle necrosis. Although elevation of creatine kinase (CK) levels was seen in all patients, a higher degree of elevation was associated with worse outcomes. Elevations of CK in influenza infection are associated with longer duration of mechanical ventilation (15 days vs 11 days, *P* < .001) and longer median ICU length of stay (13 days vs 8 days, *P* = .01).^167^ Seven patients had muscle biopsies obtained in their evaluation—one was normal and the other six demonstrated varying degrees of necrosis, regeneration, and edema—all of which can be consistent with a diagnosis of rhabdomyolysis. Of four patients with myoglobin tested in urine, all were positive.

Rhabdomyolysis can lead to renal failure by tubular obstruction from excess myoglobin, direct tubular injury, or vasoconstriction of renal blood vessels. Of the 27 reported cases, 16 patients (59%) had renal failure and 12 (44%) ultimately required renal replacement therapy (RRT). Outcomes for the twelve patients who required RRT were generally good with 10 (83%) recovering kidney function without the need for RRT beyond hospitalization, 1 died, and another required RRT beyond discharge.

While in vitro studies have demonstrated the ability of influenza to infect human skeletal muscle, this has not been reliably demonstrated in vivo as there have been only two cases in which influenza virus has been isolated from the muscle tissue of patients with rhabdomyolysis.^164^, ^168^, ^169^, ^170^ Histopathologic evaluation of affected muscle argues against an inflammatory myopathy as biopsies reveal patchy necrosis with little inflammatory infiltration.^171^, ^172^ Early recognition of this influenza‐associated complication is necessary to institute aggressive therapy for myositis‐related complications including rhabdomyolysis and/or compartment syndrome.

### 2.4 Ocular manifestations of influenza infection

Influenza‐associated ocular disease can result from direct conjunctival invasion by influenza virus and presents most commonly as a conjunctivitis although retinopathy, uveal effusion syndrome, and optic neuritis have also been reported.^173^ Avian influenza viruses of the H7 subtype in particular, with the exception of zoonotic A(H7N9) display a prominent ocular tropism compared to other strains of influenza. The human conjunctiva expresses the alpha 2‐3 sialic acid residue which is preferentially recognized by avian influenza viruses but lacks the alpha‐2,6 sialic acid residue that is classically recognized by human influenza viruses.^174^ During an outbreak of avian A(H7N7) influenza in the Netherlands, 91% (75/82) of patients presented with conjunctivitis alone and 6% (5/82) had both conjunctivitis and influenza‐like illness.^175^, ^176^ In British Columbia and Mexico, during outbreaks of highly pathogenic avian A(H7N3) infection in poultry, two patients in each country were diagnosed with infection after presenting with conjunctivitis.^177^, ^178^ In a study of 194 individuals who were actively involved in the culling of infected poultry during the A(H7N7) outbreak in the Netherlands, oseltamivir prophylaxis significantly reduced the risk of infection per farm visit from 0.145 (95% CI 0.078‐0.233) to 0.031 (95% CI 0.008‐0.073), suggesting that oseltamivir may have efficacy against influenza‐associated conjunctivitis.^179^ Similar results were found in murine ocular challenge models in which oseltamivir was shown to reduce A(H7N7) and A(H7N3) viral replication in both ocular and respiratory tissue.^180^

Conjunctivitis has also been reported in association with the A(H1N1)pdm09 virus.^174^ In a study of 89 patients with H1N1 infection, 58 (65%) presented with conjunctivitis, 7 patients (8%) presented with uveal effusion syndrome—a unilateral red painful eye associated with severe visual loss—and another 3 (3%) presented with optic neuritis.^173^ Both the uveal effusion syndrome and the optic neuritis responded to treatment with corticosteroids. More severe ocular manifestations have been reported in association with influenza A(H1N1)pdm09 infection—two cases of acute retinitis and a case of bleeding follicular conjunctivitis.^181^, ^182^, ^183^ All three patients recovered their vision although one did have persistently impaired color perception. In the patient with bleeding follicular conjunctivitis, viral RNA was detected by RT‐PCR in the affected eye and in one of the retinitis cases, the patient had vitreous antibodies to A(H1N1).

The ophthalmologic complications of influenza are reported variably and may be strain‐specific based on the evidence reviewed. However, understanding of these complications is likely limited by under‐recognition and under‐reporting.

### 2.5 Renal complications of influenza

Influenza infection can also affect renal function through a number of complications including acute kidney injury (AKI), acute glomerulonephritis, minimal change disease, and acute tubulointerstitial nephritis (ATN).

#### 2.5.1 Acute kidney injury (AKI)

Observational studies suggest the incidence of influenza‐associated AKI ranges from 18% to 66% in patients cared for in an ICU setting.^184^, ^185^, ^186^ The degree of renal failure can be quite severe, as 8%‐22% of patients ultimately require RRT during their hospitalization.^187^ The direct role of influenza vs critical illness in general as the etiology of AKI remains unclear, as this rate is similar to RRT utilization among critically ill patients without influenza. Risk factors for the development of AKI include obesity, presence of chronic kidney disease prior to illness, older age, and increased severity of illness at admission as determined by various scoring systems including the Acute Physiology and Chronic Health Evaluation II (APACHE II) and Sequential Organ Failure Assessment (SOFA).^188^, ^189^ Additionally, the association between AKI and mortality in influenza illness is conflicting, although analysis of the largest prospective cohort of A(H1N1)pdm09‐infected patients suggests that only severe AKI, category III by the Acute Kidney Injury Network (AKIN), is independently associated with mortality.^186^

The underlying pathogenic mechanism of renal injury in influenza infection is likely multifactorial. Aside from rhabdomyolysis‐mediated kidney injury, possible mechanisms include decreased renal perfusion secondary to hypovolemia or the vasodilatory state of sepsis frequently associated with severe influenza infection, potentially leading to an acute tubular necrosis (ATN). One study of autopsy findings in five A(H1N1)pdm09 cases found evidence of ATN in all patients.^190^ Similarly, in a study of 21 patients who died with A(H1N1)pdm09 infection in Brazil, all patients demonstrated mild‐to‐moderate ATN; four of those patients also had myoglobin pigment in the tubules consistent with rhabdomyolysis, and a fifth had evidence of thrombotic angiopathy.^191^ Direct viral injury to the kidneys is another postulated mechanism of disease, but there is limited evidence to support this. Influenza A(H1N1)pdm09 virus has been detected in the cytoplasm of glomerular macrophages in the kidneys of 4 of 5 patients in one post‐mortem study; however, this may suggest circulating virus or simply the presence of viral genetic material in macrophages rather than direct invasion.^190^ There is only one report of a patient with evidence of viral shedding in urine.^192^ Renal complications in the setting of influenza infection, whether an exacerbation of an underlying condition or a novel consequence of the infection, remain poorly defined and warrant further investigation.

### 2.6 Hepatic complications of influenza

Hepatic complications of influenza are rarely reported, yet recent reports suggest that liver injury occurs in the setting of influenza infection.^193^, ^194^, ^195^ In an uncontrolled human challenge model of influenza infection, 4 of 15 human subjects experimentally inoculated with influenza developed elevated blood transaminase levels (greater than threefold the upper limit in two subjects).^196^ Liver injury may be strain‐specific as a greater proportion of 97 patients with A(H1N1)pdm09 infection had elevated AST and ALT (25.78% and 26.31%, respectively) compared with 86 patients infected with 2008 seasonal influenza virus‐infected (18.6% and 7.36%, respectively).^193^ Additionally, histopathologic evidence of centrizonal hemorrhagic necrosis was also reported in 78% of patients who died following A(H1N1)pdm09 virus infection.^197^, ^198^ However, liver damage may be a marker of disease severity as liver function tests including transaminases, bilirubin, and GGT were also associated with duration of hospitalization, hypoxia, and CRP.^193^ Similarly, transaminase elevations have been more commonly reported in severe cases of influenza including 60% (6/10) of patients infected with highly pathogenic avian A(H5N1) and in 66% (73/111) of patients with A(H7N9) infection.^198^, ^199^

Post‐mortem study of three fatal cases of A(H1N1)pdm09 virus infection demonstrated viral particles in the endothelial cells, sinusoidal epithelial cells, and hepatic macrophages.^200^ Influenza virus has also been isolated and cultured from the liver of a patient that died of influenza A infection.^201^ However, evidence of viral invasion in hepatocytes has not been reliably demonstrated. Alternatively, hepatitis may develop as the result of collateral damage during a systemic immune response against a non‐hepatotropic viral infection or in response to drugs that are administered during treatment. This was supported in a small study of 11 patients infected with avian A(H7N9) virus infection who demonstrated a significant correlation between AST levels and the Th2 cytokines IL‐4 and IL‐9.^202^ The absence of influenza virus antigen in hepatocytes and the correlation between elevated liver function tests and systemic markers of inflammation including CRP suggests that the underlying pathogenesis of influenza‐associated hepatic disease is likely a result of systemic inflammation.^193^

Influenza has also been implicated as the trigger for development of acute cellular rejection in patients who have undergone orthotopic liver transplant. In two liver transplant patients infected with influenza A(H1N1)pdm09 virus, both developed acute cellular rejection (ACR) immediately following infection and demonstrated a delayed response to corticosteroids.^203^

Liver injury due to influenza infection, possibly secondary to systemic inflammation mediated by viral infection, appears to be present in a percentage of cases suggesting that liver enzymes should be monitored closely.

### 2.7 Hematologic complications of influenza

Influenza infection is associated with a variety of hematologic complications including thromboembolic disease, thrombotic thrombocytopenic purpura (TTP), hemolytic‐uremic syndrome (HUS), and hemophagocytic syndrome (HPS).

#### 2.7.1 Thromboembolic disease

Post‐mortem studies of patients who died from influenza infection have offered conflicting evidence about the association between influenza infection and thromboembolic disease likely due to differences in study design. In eight cases of fatal influenza A(H1N1), a higher incidence of pulmonary thrombi (75%) was found in comparison with the reported frequency in intensive care unit patients at autopsy (5%).^204^ However, in a nested case‐control study of patients suspected to have pulmonary embolism (PE), influenza infection was not identified as an independent risk factor (adjusted OR .22; 95% CI 0.03‐1.72.^205^ Similarly, a retrospective review of 119 patients admitted with A(H1N1)pdm09 infection found that only 5.9% (7/119) had clinically significant thrombotic events, specifically DVT (3/7; 43%), PE (2/7; 29%), MI (2/7; 29%), and one aortic thrombus (1/7; 14%).^206^ However, a large French case‐control study of 1454 adults found that influenza vaccination correlates with a reduced risk of venous thromboembolic disease, including both PE and DVT (OR = 0.74, 95% CI 00.57‐0.97).^207^

#### 2.7.2 Hemolytic‐uremic syndrome (HUS) and thrombotic thrombocytopenic purpura (TTP)

Hemolytic‐uremic syndrome (HUS) and thrombotic thrombocytopenic purpura (TTP), two overlapping thrombotic microangiopathic syndromes defined by non‐immune‐mediated hemolytic anemia, thrombocytopenia, AKI, and neurologic abnormalities, have been rarely associated with influenza infection in adults. Only four patients with influenza‐associated HUS or TTP have been described including one case that occurred in a renal transplant patient and one patient who had prior TTP and experienced a recurrence in the setting of influenza infection.^208^, ^209^, ^210^, ^211^ It has been postulated that the viral neuraminidase protein unmasks the Thomsen‐Friedenreich cryptoantigen, which has previously been implicated in atypical HUS.^208^, ^212^ Alternatively, one patient with influenza‐associated TTP developed high titer antibodies against ADAMTS13 (a disintegrin and metalloproteinase with a thrombospondin type 1 motic, member 13), a protein that cleaves von Willebrand factor (vWF) multimers.^209^ The association between influenza and TTP, however, is based on a paucity of case reports and thus remains speculative.

#### 2.7.3 Hemophagocytic syndrome

Hemophagocytic syndrome (HPS) is a clinical condition characterized by activated macrophages and histiocytes leading to secretion of an extraordinary amount of cytokines and an uncontrolled phagocytosis of platelets, erythrocytes, and lymphocytes. It is an uncommon condition thought to be triggered by specific autoimmune conditions, infections, or malignancy. However, the pathologic finding may be more common than thought as one post‐mortem review of six patients who died of influenza A(H5N1) infection all had evidence of hemophagocytosis in the bone marrow on autopsy.^213^ Influenza has also been linked to the development of virus‐associated hemophagocytic syndrome in three case reports.^36^, ^41^, ^214^ Two of the patients had a prior history of an autoimmune condition that has been independently linked to HPS including one with systemic lupus erythematous and another with rheumatoid arthritis.^215^ All patients were treated with high‐dose steroids, and one was treated additionally with IVIG and all survived. The association between influenza infection and TTP and HPS provides further evidence that in addition to respiratory disease, influenza can exacerbate underlying conditions including activating or reactivating autoimmune conditions. Early recognition of the hematologic complications of influenza including TTP or HPS in influenza infection is critical to initiate specific therapeutic interventions.

### 2.8 Endocrine complications of influenza

Patients with diabetes mellitus are considered high risk for severe influenza illness by the Center for Disease Control as infectious diseases are frequently the trigger for diabetic complications including diabetic ketoacidosis (DKA) and hyperglycemic hyperosmolar non‐ketotic coma (HHNK).^216^ The association with these diabetic complications and influenza was first reported in 1970, when 14 of 29 patients (48%) admitted to a hospital in Birmingham, England, also had respiratory complaints during a local outbreak of influenza.^217^ Since then, there have been at least 4 case reports of DKA or HHNK in the setting of diagnosed influenza—3 with influenza A(H1N1) infection and one with influenza B.^218^, ^219^, ^220^ Of those patients, 3 (75%) had no previous history of diabetes and the diagnosis was unmasked in the setting of influenza infection. The type of diabetes was not identified in most of the case reports except for one patient who had undetectable serum C‐peptide levels after glucagon administration, suggesting a diagnosis of type I diabetes with no endogenous insulin secretion; however, this patient also did not have detectable anti‐GAD or anti‐IA‐2 antibodies, the presence of which can be helpful in the diagnosis of late autoimmune diabetes in adults.^219^ The relationship between diabetes and influenza infection is also supported by epidemiologic evidence from the Netherlands. Using data on national hospitalizations aggregated during time periods with increased diagnoses of influenza‐like illness in 1976 and 1978, patients were significantly more likely to die of DKA than compared to periods when influenza‐like illness rates were at baseline (25.7% vs 14.6%, *P* < .01).^221^ This study also noted that the number of hospitalizations for DKA increased by 50% in 1978 compared to the two years prior and the year after. Although evidence is lacking to support a true association between influenza infection and endocrine complication as opposed to an exacerbation of an underlying disease, a high threshold of suspicious should be maintained in assessing for complications of HHNK or DKA.

### 2.9 Limitations

The quality of the literature presented is limited as this review relies primarily on case reports, case series, and observational studies. The complications identified are also difficult to address in the context of severe systemic influenza infection as it is unclear if they are the consequence of the infection itself, systemic illness or shock in general, or an exacerbation of an otherwise underlying condition. Additionally, publication bias likely exists as a disproportionately large amount of the literature comes from the 2009 H1N1 influenza pandemic.

## 3 CONCLUSIONS

Influenza viruses are global pathogens that infect up to 20% of the world population each year and cause significant morbidity and mortality. However, the burden of influenza is largely based on the identification of well‐recognized respiratory‐related manifestations. Data presented here from an comprehensive literature review suggest that extra‐pulmonary complications, including influenza‐associated cardio‐ and cerebrovascular events, myocarditis, CNS syndromes, and rhabdomyolysis, constitute an under‐recognized burden of disease in patients infected with influenza. Influenza‐specific prevention with vaccines has been shown to reduce the risk of some of these complications but further studies are needed. Similarly, early antiviral therapy appears to reduce the risk and/or severity of certain complications. Early recognition of the extra‐pulmonary manifestations of influenza infection is critical to the initiation of therapeutic interventions and organ‐specific supportive care.

## CONFLICT OF INTEREST

SS, RH, FH, and WF report no relevant conflicts of interest.

## ETHICS COMMITTEE APPROVAL

N/A.

## APPENDIX A

### Summary of evidence considered by section

Seventy‐five articles that considered cardiac complications of influenza infection were included. Forty‐five of these papers considered influenza‐associated myocarditis, including 23 case reports, 7 case series, 10 observational studies, 3 observational autopsy studies, and 2 review articles. Twenty‐one manuscripts were reviewed regarding the association between ischemic heart disease and influenza, including 8 case‐control studies, 4 observational studies, 3 time series studies, 2 randomized control studies, 2 review articles, and 2 animal studies. Nine articles regarding stroke in influenza were reviewed, including 4 cohort studies, 3 case‐control studies, and 2 case reports.

Sixty‐nine articles that considered neurologic complications of influenza infection were included. Thirty‐nine of these papers discussed influenza‐associated encephalitis and encephalopathy, including 20 case reports, 10 observational cohort studies, 5 case series, 3 basic science articles, and 1 review. Fifteen manuscripts were reviewed regarding the association between Guillain‐Barre syndrome and influenza, including 4 case reports, 3 case series, 3 case‐control studies, 2 observational cohort studies, 2 time series studies, and 1 review article. Three studies were reviewed regarding the association between narcolepsy and influenza infection, including 1 observational cohort study, 1 review article, and 1 animal study. Similarly, three articles were reviewed regarding Reye's syndrome in the setting of influenza including 2 case series and 1 review article. Seven manuscripts discussing the association of influenza with encephalitis lethargica and Parkinson's syndromes, including 4 autopsy studies, 1 case‐control study, 1 case series, and 1 case report.

Twenty‐four manuscripts were reviewed regarding the association between influenza infection and myopathy or rhabdomyolysis. These included 14 case reports, 3 case series, 3 observational cohort studies, and 4 basic science articles.

Eleven articles were reviewed regarding ocular complications of influenza infection. Conjunctivitis was the most well‐represented complication in the literature, as there were 2 observational cohort studies, 2 case‐control studies, 3 basic science papers, 1 case series, and 1 case report. One of the case‐control studies also addressed optic neuritis as a complication, and there were three additional case reports regarding acute retinitis and bleeding follicular conjunctivitis.

Ten articles were reviewed discussing the association of acute kidney injury in influenza infection, including 6 observational cohort studies, three autopsy studies, and one case report. Although the quality of the evidence is reasonably strong, the conclusions drawn are inconsistent.

Twelve manuscripts regarding hepatic complications in influenza infection were reviewed. This included 4 autopsy studies, 4 observational cohort studies, 3 case series, 1 human virus challenge study, and 1 case‐control study. The quality of the evidence is overall weak to moderate and somewhat inconsistent as it remains unclear whether liver disease is directly related to influenza infection or whether it is simply a marker of severe infection.

Fourteen manuscripts were reviewed regarding hematologic complications of influenza infection. Four articles discussed the role of thromboembolic disease, including 2 case‐control studies, 1 cohort study, and 1 autopsy study. There were five manuscripts reviewed concerning HUS/TTP in the setting of influenza, including 4 case reports and 1 review article. As the support was mostly dependent on case reports, the evidence is weak. Five articles addressed the link between hemophagocytic syndrome and influenza infection including 4 case reports and 1 autopsy study.

Five manuscripts were reviewed regarding endocrine complications of influenza infection, including 2 case reports, 1 case series, 1 cohort study, and 1 time series.
